# Growth of Hair, Nails, and Secretions of the Body

**Published:** 1851-01

**Authors:** 


*Growth of Hair, Nails, and Secretions of the Body.*—There appears to exist a most astonishing similarity between the growth of the hair and nails, and the operation of the secretions of the body being simultaneous. The growth of the nails is singularly different, even upon the same individuals and upon the same hand and fingers. By the experiments of Berthold, it was found that they would grow upon the same finger a greater length in the summer than in the winter, also that it was much quicker upon the right hand than upon the left. The hair grows upon individuals of sixteen to twenty-four years of age about seven lines a month, which is accelerated by frequent cutting, and the reproduction goes on more rapidly during day than at night, and greater in warm than in cold weather. The quantitative formation of nail and hair coincides with the peripheric secretions, perspiration, &c., in this, that it increases in summer, and decreases in winter, whereas, the development and nutrition of the body is decreased in summer, and increased in winter, so that the weight of a man is greater in winter than in summer. The growth of the hair and the nails decreases in the night, which coincides with the decrease of the secretions, perspiration, formation of carbonic acid gas, urine, milk, bile, &c.

